# Laparoscopic antireflux surgery for refractory gastroesophageal reflux disease: long-term clinical outcomes

**DOI:** 10.1007/s13304-023-01483-x

**Published:** 2023-03-02

**Authors:** Elettra Ugliono, Fabrizio Rebecchi, Serena Mantova, Giulia Osella, Ahmed Mohammed Farid Mahmoud Hamdy Mansour, Mario Morino

**Affiliations:** 1 Department of Surgical Sciences, University of Turin, Corso A.M. Dogliotti 14, 10126 Turin, Italy; 2 Department of Mechanical and Aerospacial Engineering, Politecnico of Turin, Corso Duca Degli Abruzzi 24, 10129 Turin, Italy

**Keywords:** Gastroesophageal reflux, Fundoplication, Esophageal pH monitoring, Long-term outcomes, Predictive factors

## Abstract

Persistent symptoms despite adequate Proton Pump Inhibitors (PPI) treatment are described in up to 40% of patients with Gastroesophageal Reflux Disease (GERD). The efficacy of Laparoscopic Antireflux Surgery (LARS) in PPI non-responder patients is still unclear. This observational study aims to report the long-term clinical outcomes and predictors of dissatisfaction in a cohort of refractory GERD patients submitted to LARS. Patients with preoperative refractory symptoms and objective GERD evidence submitted to LARS between 2008 and 2016 were included in the study. Primary endpoint was overall satisfaction with the procedure, secondary endpoints were long-term GERD symptom relief and endoscopic findings. Univariate and multivariate analyses were performed to compare satisfied and dissatisfied patients, in order to identify preoperative predictors of dissatisfaction. A total of 73 refractory GERD patients who underwent LARS were included in the study. At a mean follow-up of 91.2 ± 30.5 months, the satisfaction rate was 86.3%, with a statistically significant reduction in typical and atypical GERD symptoms. Causes of dissatisfaction were severe heartburn (6.8%), gas bloat syndrome (2.8%), and persistent dysphagia (4.1%). Multivariate analysis showed that a number of Total Distal Reflux Episodes (TDRE) > 75 was a predictive factor of long-term dissatisfaction after LARS while a partial response to PPI was a protective factor against dissatisfaction. LARS guarantees a high level of long-term satisfaction for selected refractory GERD patients. An abnormal TDRE at 24 h-multichannel intraluminal impedance-pH monitoring and the lack of response to preoperative PPI were predictors of long-term dissatisfaction.

## Introduction

Gastro-esophageal reflux disease (GERD) is defined as the clinical condition that develops when abnormal reflux of gastric contents causes symptoms and/or complications. [1] Clinical manifestations of GERD include typical (heartburn, regurgitation) and atypical extraesophageal (chronic cough, hoarseness, laryngitis) symptoms. The mainstays of GERD medical treatment are antisecretory drugs, particularly Proton Pump Inhibitors (PPI). [2] However, as many as 40% of GERD patients fail to respond adequately despite appropriate PPI treatment. [3]

The management of this clinical condition, known as refractory GERD, is not trivial.

Patients with persistent symptoms potentially attributable to refractory GERD deserve further diagnostic evaluations, including upper endoscopy, esophageal manometry, and 24 h multichannel intraluminal impedance-pH (MII-pH) monitoring to confirm the diagnosis. In fact, several other disorders can be responsible for GERD-like symptoms, especially when atypical extraesophageal symptoms are prominent. [4]

Once the diagnosis is confirmed, several medical treatment options, including optimization of PPI treatments and the adjunction of other therapeutic agents can be proposed, depending on the underlying cause of PPI unresponsiveness. [5–7] However, the overall efficacy of these treatments is weak. [8]

Laparoscopic Antireflux Surgery (LARS) has been advocated for the treatment of refractory GERD. However, while LARS is highly effective for patients with typical symptoms and good response to PPI, the results of surgical treatment for refractory GERD are less clear. [9, 10] Therefore, this study aims to report the long-term results of LARS for refractory GERD, and to identify predictors of long-term dissatisfaction related to the procedure.

## Materials and methods

Data from patients who underwent LARS at our Institution for refractory GERD were collected. “Refractory” GERD patients were adult patients with persistence of GERD symptoms despite adequate PPI treatment and objective evidence of pathological GERD at instrumental examinations. Indications for surgery were refractory patients with either endoscopic evidence of GERD complications or MII-pH findings of pathological reflux. Exclusion criteria were patients with good response to PPI treatment, large hiatal hernia (> 5 cm), revisional surgery, and primary surgery with open approach.

All patients underwent upper endoscopy preoperatively to rule out the presence and degree of esophagitis according to the Los Angeles classification, the presence and extent of Barrett’s esophagus, or other eventual mucosal abnormalities requiring endoscopic biopsies. [11]

Conventional esophageal manometry was performed to assess the functionality of the lower esophageal sphincter (LES), to exclude major esophageal dysmotility disorders, and to allow the correct positioning of the MII-pH catheter.

Preoperative ambulatory MII-pH monitoring (Sleuth; Sandhill Scientific INC, Highland Ranch, CO) off medical therapy was performed in all cases. Reflux episodes were categorized into acid, weakly acidic, and weakly alkaline, depending on their chemical characteristics. Proximal reflux episodes were defined as those reaching the electrodes located 15 cm from the upper limit of the LES. MII-pH pathological cut-off values considered were those described by Zerbib et al. in a cohort of 72 healthy subjects. Thresholds for defining “abnormal” number of reflux episodes were > 75 for distal total, > 50 for distal acid, > 33 for distal weakly acidic, and > 30 for proximal reflux episodes. [12] Acid Exposure Time (AET) was considered abnormal when > 4%. [13] Symptom Index (SI) and Symptom Association Probability (SAP) were calculated and considered positive for values ≥ 50% and ≥ 95%, respectively. [14, 15]

Furthermore, in case of predominant extraesophageal symptoms, patients were evaluated by otorhinolaryngologist and pulmonary specialists, in order to exclude other possible etiologies.

### Surgical procedures

All the surgical procedures were performed with laparoscopic approach by two experienced laparoscopic surgeons (MM, FR). The surgical techniques has been described elsewhere. [16, 17] In brief, after complete esophageal distal mobilization, a primary posterior cruroplasty with non-absorbable stitches was performed in case of an enlarged hiatus. In patients undergoing total fundoplication, a 360° wrap was created according to DeMeester’s criteria (short and floppy) with the anterior aspect of the stomach with three non-absorbable stitches. In patients undergoing posterior partial fundoplication, a 270° wrap was performed with three esophagogastric stitches on the left and three esophagogastric stitches on the right aspects of the esophagus. Total fundoplication was the standard procedure, while Toupet fundoplication was performed in case of preoperative dysmotility at esophageal manometry.

### Follow-up

All patients were followed in the outpatient clinic at 1, 3, 6, 12 months and annually thereafter. For the purposes of this study, all patients were contacted to ask for consent to enter the study and undergo the clinical questionnaire.

Overall satisfaction was measured using a 10-point Likert scale. Satisfaction with the procedure was defined as a score > 7, while dissatisfaction as < 5. For intermediate scores (5–7), patients were considered satisfied if they answered “yes” to whether they would choose to undergo surgery again.

### Endpoint and definition of the variables

The primary endpoint was the assessment of long-term overall satisfaction after LARS. Secondary endpoints were long-term GERD symptoms and endoscopic findings after LARS, and the identification of causes and predictive factors for dissatisfaction related to the procedure.

Parameters included in the univariate analyses were: demographic, preoperative typical symptoms, atypical symptoms and dysphagia, endoscopic findings, type of wrap, and MII-pH monitoring.

### Statistical analysis

Continuous variables were reported as mean ± standard deviation (SD) while categorial data were reported as percentages.

Univariate analyses were performed using the t Student test for normally distributed continuous variables, chi square test applying Fisher’s exact test when appropriate for categorical variables, and Mann–Whitney test for not normally distributed data. We performed a logistic regression with backward stepwise selection: we included in the multivariate analysis all the variables with a significance level of 0.15 at univariate analysis, and a significance level of 0.15 was required for the variable to remain in the model. A p-value < 0.05 at multivariate analysis was considered statistically significant. All the analyses were performed using “Stata” software statistical program (version 17.0).

## Results

**Fig. 1:**
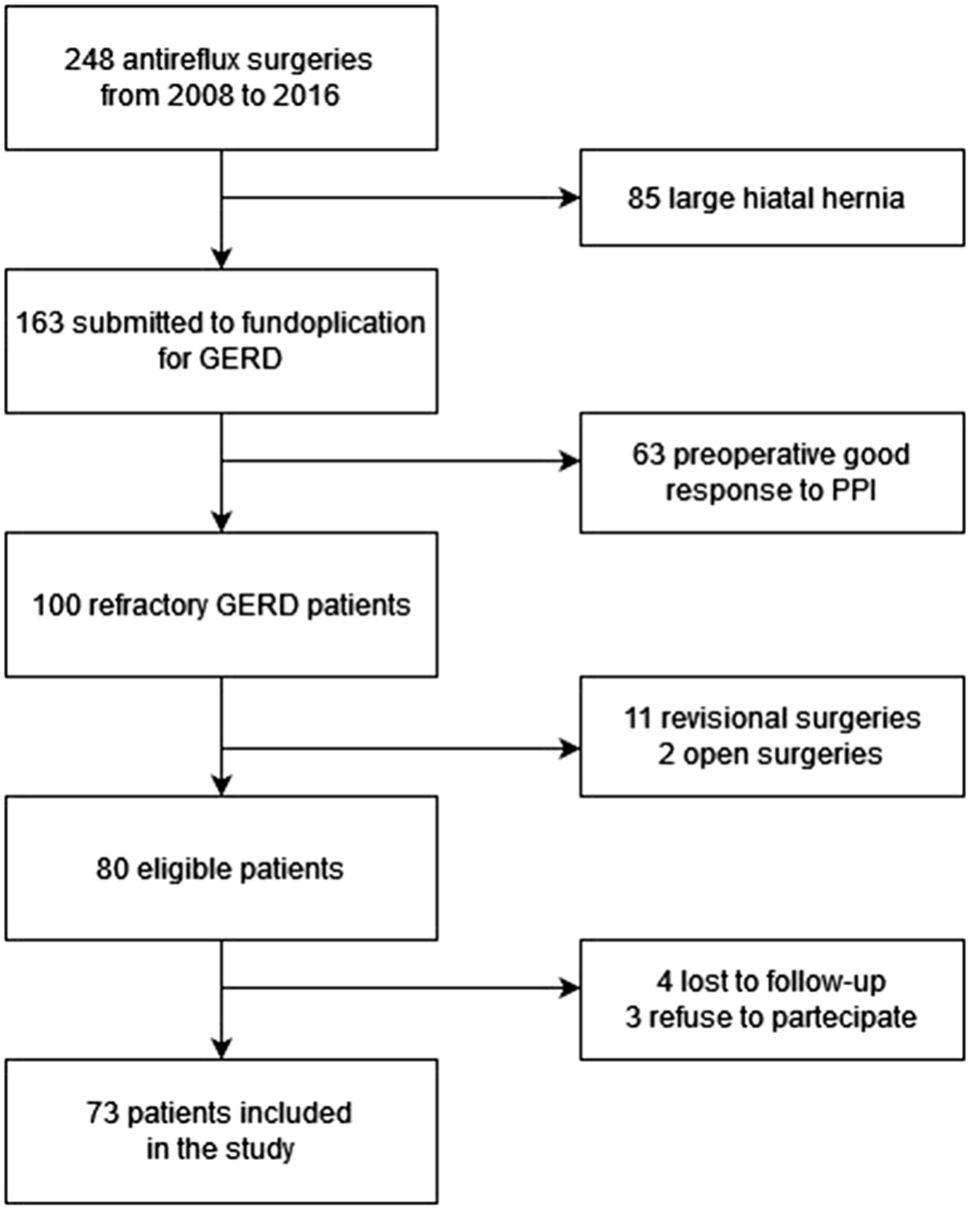
Flowchart of the included patients

A total of 248 patients underwent LARS at our Institution between January 2008 and December 2016. Of these, 85 patients had large hiatal hernias, while 63 patients had GERD with good response to PPI treatment, therefore were excluded. A total of 73 patients met all the inclusion criteria and were considered for the study. Figure 1 shows the flowchart of the included patients.

The mean age at surgery was 48.0 ± 13.0 years, 22 patients (30.1%) were female, while 51 (69.9%) were male. The median duration of symptoms was 96.9 ± 77.9 months. Before surgery, the main symptoms experienced by patients were heartburn (80.8%), regurgitation (65.8%), and atypical symptoms (53.4%). Table 1 summarizes the baseline characteristics of the patients.

**Table 1:** Baseline characteristics of included patients

	N = 73
Age [years] (mean; SD)	48.0 ± 13.0
Weight [kg] (mean; SD)	75.4 ± 11.3
BMI [kg/m^2^] (mean; SD)	25.7 ± 3.0
Preoperative smoking (n; %)	13 (17.8)
Preoperative symptoms	
Only typical symptoms	34 (46.6%)
Only atypical symptoms	15 (20.5%)
Mixed typical and atypical symptoms	24 (32.9%)
ASA score (n; %)	
1	20 (27.4)
2	48 (65.8)
3	5 (6.8)
4	0 (0)
Esophagitis (n; %)	34 (46.6)
Grade 1	19 (55.9)
Grade 2	11 (32.4)
Grade 3	2 (5.9)
Grade 4	2 (5.9)
HP infection (n; %)	7 (9.6)
Barrett’s esophagus (n; %)	16 (21.9)
Short barrett	11 (68.8)
Long barrett	5 (31.2)
Small hiatal hernia (n; %)	46 (63.0)
LES pressure [mmHg] (mean; SD)	7.2 ± 1.8
Minor dysmotility (n; %)	20 (27.4)
Number of distal reflux episodes (mean, SD)	
Acid	54.4 ± 31.6
Weakly acidic	30.2 ± 19.7
Total	84.5 ± 43.1
Number of proximal reflux episodes (mean; SD)	44.9 ± 25.9
DeMeester’s score (mean, SD)	37.4 ± 30.5
Acid exposure time (mean; SD)	8.1 ± 6.3
SAP positivity (n; %)	68 (93.2)
SI positivity (n; %)	65 (89.0)

*BMI* Body Mass Index, *ASA* American Society of Anesthesiologist, *HP* Helicobacter Pylori, *LES* Lower Esophageal Sphincter, *SAP* Symptom Association Probability, *SI*Symptom Index

### Intraoperative data

The mean operative time was 65.1 ± 17.6 min. All the procedures were completed laparoscopically with no need for conversions to open surgery. A total of 53 (72.6%) patients underwent total fundoplication, while 20 (27.4%) underwent posterior partial fundoplication. Associated cholecystectomy was performed in 3 (4.1%) patients in case of symptomatic cholelithiasis. There were no intraoperative or postoperative complications. Mortality was 0%. The mean length of stay was 2.6 ± 0.9 days.

### Follow-up results

At a mean follow-up of 91.2 ± 30.5 months, a total of 63 (86.3%) patients were satisfied with the surgical procedure, with a median overall satisfaction of 9 out of 10 (IQR 7–10).

**Fig. 2:**
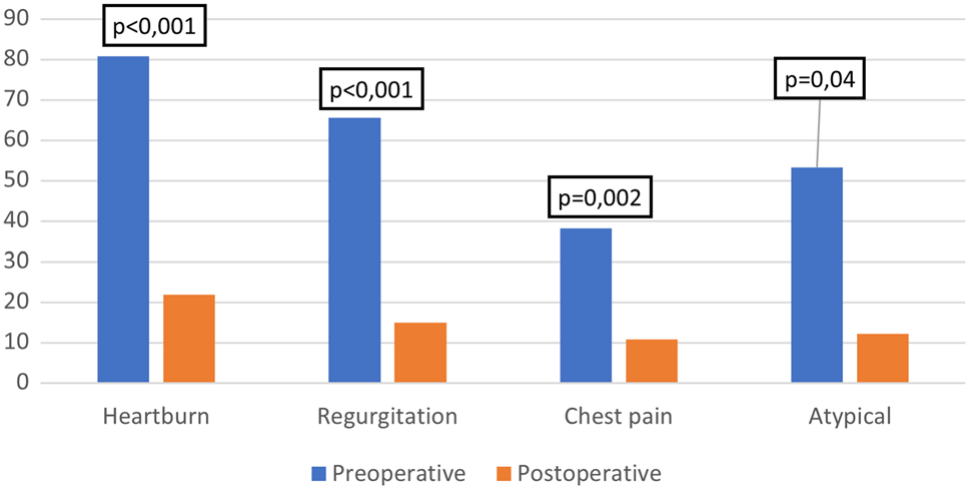
Comparison between preoperative and postoperative symptoms

At long-term follow-up there was a statistically significant reduction of GERD symptoms compared to the preoperative period, with a decreased rate of heartburn (from 80.8 to 21.9%, p < 0.001), regurgitation (from 65.7 to 15.1%, p < 0.001), chest pain (from 38.3 to 10.9%, p = 0.002) and atypical symptoms (from 53.4% to 12.3%, p = 0.04). (Fig. 2).

Postoperative dysphagia was present in 17 (23.3%) patients, described as mild and occasional in 14 (19.1%) and severe in 3 (4.1%), while gas bloat syndrome was reported to be mild by 8 (10.9%) and severe by 2 (2.8%) patients. All patients with complaints of postoperative dysphagia were investigated with objective examinations, through upper endoscopy and radiological series with contrast medium, to exclude the possibility of a tight wrap or anatomical distortions of the wrap) that could be responsible for the symptoms.

Long-term endoscopic evaluation was available for 59 (80.8%) patients after a mean of 68 ± 37.6 months. In 44 patients (74.5%), there were no signs of inflammation of the esophageal mucosa. Of the 16 patients with preoperative Barrett’s esophagus, the long-term endoscopic evaluation was obtained in 10 (62.5%), with evidence of intestinal metaplasia resolution in 5 patients with short Barrett.

### Predictors of dissatisfaction

Dissatisfaction with the procedure occurred in 10 (13.7%) patients due to severe heartburn in 5 (6.8%), gas bloat syndrome in 2 (2.8%), and persistent dysphagia in 3 (4.1%).

By univariate analyses, preoperative factors considered for multivariate analysis were heartburn (OR 0.28, 95%CI 0.07–1.10 p = 0.07), partial response to preoperative PPI (OR 0.33 95%IC 0.09–1.23 p = 0.10), Nissen fundoplication (OR 0.31 95%CI 0.08–1.15 P = 0.08), number of Total Distal Reflux Episodes (TDRE) (OR 11.25 95%CI 1.70-.) p = 0.007), and number of weakly acidic distal reflux episodes (OR 2.60 95%CI 0.70–9.55, p = 0.15). (Table 2) By multivariate analysis, TDRE was predictive factor of dissatisfaction after LARS, while a partial response to PPI was a protective factor against dissatisfaction. (Table 3).

**Table 2:** Univariate analyses for predictors of dissatisfaction

	Univariate analysis	
Variable	OR	P
Age > 65 years	2.37 (0–12.9)	0.32
BMI > 25	1.75 (0.48–6.39)	0.41
Preoperative smoking	0.47 (0–3.24)	0.48
Preoperative heartburn	0.28 (0.07–1.10)	0.07
Preoperative chest pain	1.08 (0.29–3.99)	0.90
Preoperative regurgitation	1.25 (0.31–4.87)	0.76
Preoperative dysphagia	1.05 (0–7.77)	0.96
Preoperative atypical symptoms	1.36 (0.37–4.95)	0.65
Partial response to PPI	0.33 (0.09–1.23)	0.10
Distal acid reflux episodes	1.54 (0.42–5.62)	0.52
Distal WAC reflux episodes	2.60 (0.70–9.55)	0.15
7Distal total reflux episodes	11.25 (1.70-.)	0.007
DeMeester’s score	1.47 (0.31-.)	0.64
AET > 4	1.37 (0.34–5.35)	0.66
Proximal reflux episodes	1.86 (0.40-.)	0.45
Esophagitis	1.75 (0.48–6.39)	0.41
Barrett esophagus	0.35 (0–2.40)	0.32
Nissen fundoplication	0.31 (0.08–1.15)	0.08

*OR* Odds Ratio, *BMI* Body Mass Index, *PPI* Proton Pump Inhibitors, *WAC* Weakly Acidic, *AET* Acid Exposure Time

**Table 3:** Multivariate analyses for predictors of dissatisfaction

Variable	OR	Complete model		OR	Reduced model	
		Regression coefficient	P		Regression coefficient	P
Preoperative heartburn	0.52 (0.09–3.17)	− 0.63	0.48			
Partial response to PPI	0.22 (0.03–1.41)	− 1.48	0.11	0.15 (0.03–0.75)	− 1.85	0.02
Distal WAC reflux episodes	0.38 (0.06–2.46)	− 0.96	0.31			
Distal total reflux episodes	33.7 (2.36–480.8)	3.51	0.009	20.3 (2.12–194.0)	3.01	0.009
Nissen fundoplication	0.46 (0.08–2.45)	− 0.77	0.36			

*OR* Odds Ratio, *PPI* Proton Pump Inhibitors, *WAC* Weakly Acidic

## Discussion

Laparoscopic fundoplication provides an effective and durable GERD symptom relief comparable to long-term medical treatment, especially in patients with typical symptoms and good response to PPI. [9, 18, 19] LARS consists of the creation of a wrap that acts as a mechanical barrier to prevent the refluxate of the gastric content independently from its chemical characteristics. Therefore, it has been suggested also for the treatment of refractory GERD. However, the results of LARS in refractory GERD patients are unclear, and the optimal treatment for this subgroup of patients remains uncertain. [20, 21]

Recently, Spechler et al. performed a randomized clinical trial comparing LARS (27 patients), active medical treatment (omeprazole plus baclofen, 25 patients), and control medical treatment (omeprazole plus placebo, 26 patients) for refractory heartburn. The authors found that, at 1-year follow-up, LARS was associated with a significantly higher satisfaction compared to the active and control medical group (67% vs. 28% vs. 12% respectively, p < 0.001). [22] However, satisfaction was considerably lower than the incidence of success after LARS reported in observational studies of non-refractory GERD patients. [23]

Several mechanisms can explain the lack of response of GERD symptoms to acid-reducing medications, such as inadequately controlled acid reflux, predominant weakly acidic/non-acid reflux, or overlap with other esophageal or extra-esophageal non-GERD conditions. [2] The diagnosis of “true” refractory GERD, therefore, is not easy and requires a systematic evaluation, including endoscopy, esophageal manometry, and MII-pH monitoring. [24] Specifically, MII-pH monitoring, enabling the evaluation of the number, chemical characteristics, composition, and extent of reflux episodes and their correlation with symptoms, is useful both to characterize the underlying main type of reflux, and to distinguish refractory GERD patients from alternative diagnoses unrelated to GERD. [25]

This study, aimed to evaluate the clinical outcomes of LARS in the treatment of refractory GERD, demonstrated the durable effects of this procedure in controlling GERD symptoms, with an overall satisfaction rate of 86% at long-term follow-up. These results align with those reported by other authors, describing excellent LARS outcomes that are maintained up to 20 years after surgery in non-refractory GERD patients. [26, 27] Furthermore, LARS resolved effectively both typical and extraesophageal atypical symptoms, that are generally associated with lower rates of improvement and resolution after surgery. [28]

The successful outcomes of LARS largely depend on a careful preoperative assessment and rigorous patient selection. [29, 30] This is particularly true for PPI unresponsive patients, since GERD-like typical and atypical symptoms of different etiologies can often be mistaken for refractory GERD. Therefore, before considering LARS, a clear demonstration that persistent GERD symptoms are truly reflux-related is mandatory.

The results of this study indicate that preoperative endoscopy and MII-pH monitoring allowed a precise selection of patients presenting with refractory GERD, even in case of extraesophageal symptoms, leading to a remarkable satisfaction rate after LARS.

However, while most patients experienced significant relief of symptoms after surgery, a small subset of patients is dissatisfied with the procedure due to the persistence of symptoms or the onset of side effects such as dysphagia and gas bloat syndrome. Several demographic, clinical, and instrumental parameters have been investigated to identify specific factors that could influence surgical outcomes [31–33].

In this study, we performed univariate and multivariate analyses, including patient characteristics, preoperative symptoms, and instrumental parameters, particularly MII-pH monitoring variables, in order to identify factors associated with postoperative procedure-related dissatisfaction. We found that a partial response to PPI was a protective factor against dissatisfaction, while an abnormal TDRE at preoperative MII pH monitoring off PPI was a predictive factor of dissatisfaction at long-term follow-up after LARS.

A reason for this is not entirely clear, since TDRE is an easily quantifiable MII-pH metric, but its clinical relevance is incompletely elucidated. It could be argued that a higher TDRE documents a more severe degree of GERD, since there is evidence that a higher TDRE correlates with increasing grades of esophagitis and Barrett’s esophagus. [34, 35] Therefore, refractory GERD patients with evidence of an elevated number of TDRE at preoperative MII-pH monitoring may be offered LARS bust should be aware of a potential higher risk of dissatisfaction after the procedure compared to patients with normal TDRE.

Future efforts should be directed at confirming our preliminary results and elucidating the clinical implications of abnormal MII-pH parameters on medical and surgical outcomes, tailoring GERD treatments according to patient characteristics.

This study has limitations that deserve comments. The primary endpoint of our study was the analysis of overall satisfaction, which is a subjective outcome. To date, there are no validated tools capable of taking into account the whole spectrum of possible outcomes of the procedure, including improvement in quality of life, typical and atypical symptoms, and the occurrence of dysphagia and gas bloat syndrome. Besides, we did not assess the role of emerging techniques, such as high-resolution manometry, that were not available at the time of the surgical procedures.

## Conclusion

LARS for selected refractory GERD patients guarantees a high level of overall satisfaction, that is maintained in the long-term follow-up. Preoperative MII-pH monitoring is a useful complementary tool to allow a correct selection of PPI unresponsive patients before LARS, allowing remarkable long-term satisfaction. An elevated abnormal number of TDRE was the only predictor of long-term dissatisfaction after the procedure.

## Data Availability

The data that support the findings of this study are available from the corresponding author upon reasonable request.
